# A short history of the development of mathematical models of cardiac mechanics

**DOI:** 10.1016/j.yjmcc.2018.11.015

**Published:** 2019-02

**Authors:** Steven A. Niederer, Kenneth S. Campbell, Stuart G. Campbell

**Affiliations:** a Biomedical Engineering, Kings' College London, London, UK; b Department of Physiology and Division of Cardiovascular Medicine, University of Kentucky, Lexington, USA; c Departments of Biomedical Engineering and Cellular and Molecular Physiology, Yale University, New Haven, USA

## Abstract

Cardiac mechanics plays a crucial role in atrial and ventricular function, in the regulation of growth and remodelling, in the progression of disease, and the response to treatment. The spatial scale of the critical mechanisms ranges from nm (molecules) to cm (hearts) with the fastest events occurring in milliseconds (molecular events) and the slowest requiring months (growth and remodelling). Due to its complexity and importance, cardiac mechanics has been studied extensively both experimentally and through mathematical models and simulation.

Models of cardiac mechanics evolved from seminal studies in skeletal muscle, and developed into cardiac specific, species specific, human specific and finally patient specific calculations. These models provide a formal framework to link multiple experimental assays recorded over nearly 100 years into a single unified representation of cardiac function. This review first provides a summary of the proteins, physiology and anatomy involved in the generation of cardiac pump function. We then describe the evolution of models of cardiac mechanics starting with the early theoretical frameworks describing the link between sarcomeres and muscle contraction, transitioning through myosin-level models to calcium-driven systems, and ending with whole heart patient-specific models.

## 1 Introduction

### 1.1 Cardiac physiology

The central function of the heart is to circulate blood throughout the body. Blood flow results from the combined action of the cardiac valves and rhythmic contractions of the heart's four chambers. Pumping movement by the chambers is made possible by a high density of contractile cells within the chamber walls. These muscle cells or cardiomyocytes are in turn packed with dense arrays of contractile structures known as sarcomeres.

The sarcomere is the smallest functional unit of contraction. It is comprised of so-called thick and thin filaments that are arranged in large parallel arrays [1]. Each thick filament is typically surrounded by six thin filaments in a repeating pattern that forms a highly organized lattice. With the two filament systems thus interdigitated, they can slide past one another [2]. Their proximity also allows the myosin motor proteins that protrude from the thick filament to attach to binding sites on the thin filament. When such an attachment forms, it creates a mechanical link between thick and thin filaments known as a crossbridge [[3], [4], [5]]. Once a crossbridge forms, myosin undergoes an ATP-driven molecular rearrangement called a powerstroke that distorts the crossbridge and creates a pulling force that tends to slide the thin and thick filaments past each other [6,7]. After the powerstroke and binding of a new ATP molecule to the myosin head, it releases from the thin filament. As sufficient numbers of myosin heads cyclically attach to, pull on, and detach from thin filaments, the sarcomere contracts.

As a pulsatile pump, the heart requires a system for precise activation and deactivation of contractile activity in the sarcomeres. A heart beat is the result of an electrical signal that passes through the myocardium, triggering within each cell a transient increase in Ca^2+^ concentration [8]. Ca^2+^ ions are the essential control signal that activates contraction at the level of the sarcomere [9]. The Ca^2+^ signal is transduced by the thin filaments [10], which contain proteins that form a sensitive and highly tuned allosteric signalling complex (reviewed in [11]). The thin filaments are composed of an actin filament backbone decorated with accessory proteins that include tropomyosin and the three subunits of troponin (C, I, and T) [12]. Tropomyosin is an elongated coiled-coil dimer that spans seven actin subunits [13]. Under conditions of low Ca^2+^, tropomyosin blocks myosin binding sites on actin and prevents crossbridge formation. This is the relaxed state of the sarcomere. When Ca^2+^ is present, it binds to a single site on troponin C and triggers a series of conformational changes in the troponin-tropomyosin-actin complex. The end result is movement of tropomyosin azimuthally on the surface of actin to expose myosin binding sites, and contraction is initiated [14]. The Ca^2+^ transient recovers as Ca^2+^ is removed from the cytosol by the sarcoplasmic reticulum Ca^2+^ -ATPase and the membrane sodium/calcium exchanger. Ca^2+^ recovery starts prior to peak tension. As the Ca^2+^ transient falls, Ca^2+^ dissociates from troponin C, in turn allowing tropomysoin to return to its blocked position, inhibiting crossbridge binding.

Macroscopically, sarcomeres constitute a bulk material with anisotropic, time-varying mechanical properties [15]. The activity of myosin ATPase makes the sarcomere a unique class of ‘active’ material, capable of producing mechanical work. Components within the sarcomere also provide passive mechanical elements that contribute to the material properties of myocardium, such as the giant protein titin [16]. Titin acts as a molecular spring in parallel with the freely sliding thick and thin filaments, providing mechanical stability to the sarcomere during relaxation.

Both passive and active components of the sarcomeres are linked to the cell exterior through a host of additional structural proteins [17] that connect cardiomyocytes to the extracellular matrix and adjacent cells. The brick-shaped cardiomyocytes are arranged with their long axes oriented along a common axis to form the fibres and sheets of contractile tissue that comprise the chamber walls [18]. Hence, sarcomeres are mechanically linked across scales to the fluid loads within the heart. The activity of myosin is thereby capable of exerting mechanical forces on blood to generate ejection during systole. By the same token, blood filling the chambers during diastole acts to stretch the relaxed sarcomeres and modify their behaviour – as sarcomere length changes, the configuration of its constituent proteins is altered [19] and contraction force is modulated such that contractions initiated at longer sarcomere lengths produce more force [[20], [21], [22], [23]]. This phenomenon, known at the ventricular level as the Frank-Starling relationship, is often referred to as length-dependent activation, and has been studied experimentally by numerous groups. The precise mechanisms remain unclear (see [24,25]) but may include length-dependent changes in myofibrillar lattice spacing [26], strain-sensitive interactions within and between troponin and tropomyosin [27], and interactions between titin and the thick and thin filaments [28]. A new hypothesis that has been developed in the last few years suggests that length-dependent activation is caused by stretch-induced changes in thick filament structure [[29], [30], [31], [32]].

**Fig. 1:**
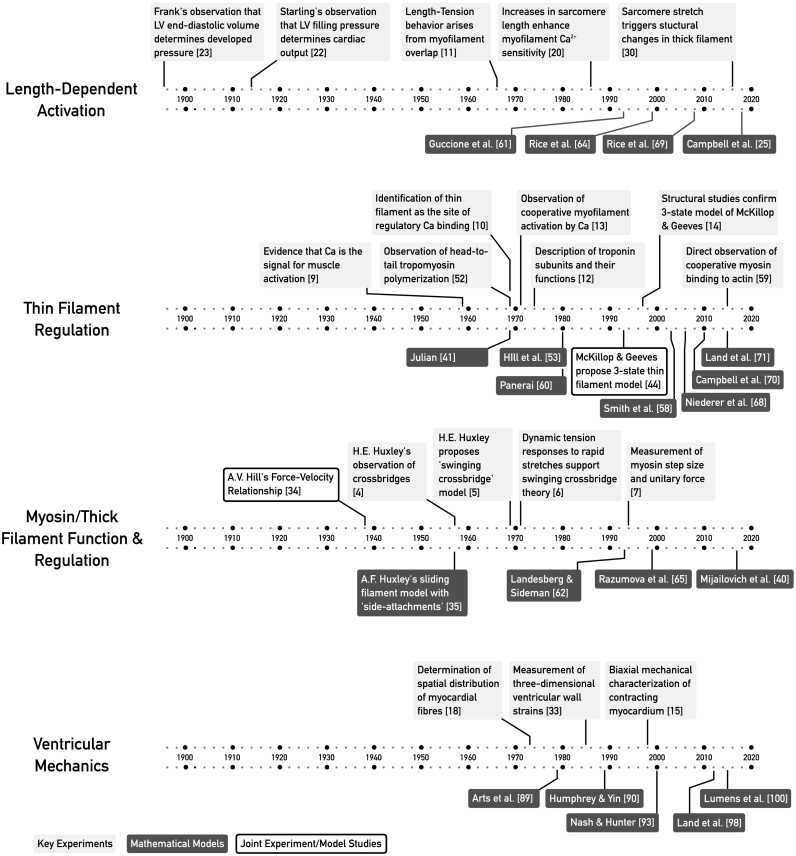
Functional properties of isolated myocardium. Panels show examples (in schematic form) of: A) the force-velocity relationship, B) isometric twitch, C) length-dependent changes in Ca^2+^-dependent force, D) force response to step length changes, and E) sinusoidal analysis.

Ultimately, the proteins of the sarcomere and their structural arrangement impart dependencies of time, Ca^2+^, sarcomere length, rate of sarcomere length change (i.e. velocity), and load to the mechanical properties of the myocardium. These relationships have been painstakingly elucidated through many decades of biomechanical testing of cardiac tissue preparations that include whole ventricles, intact trabeculae or papillary muscles, individual cardiomyocytes, and ultimately isolated myofibrils (Fig. 1).

**Fig. 2:**
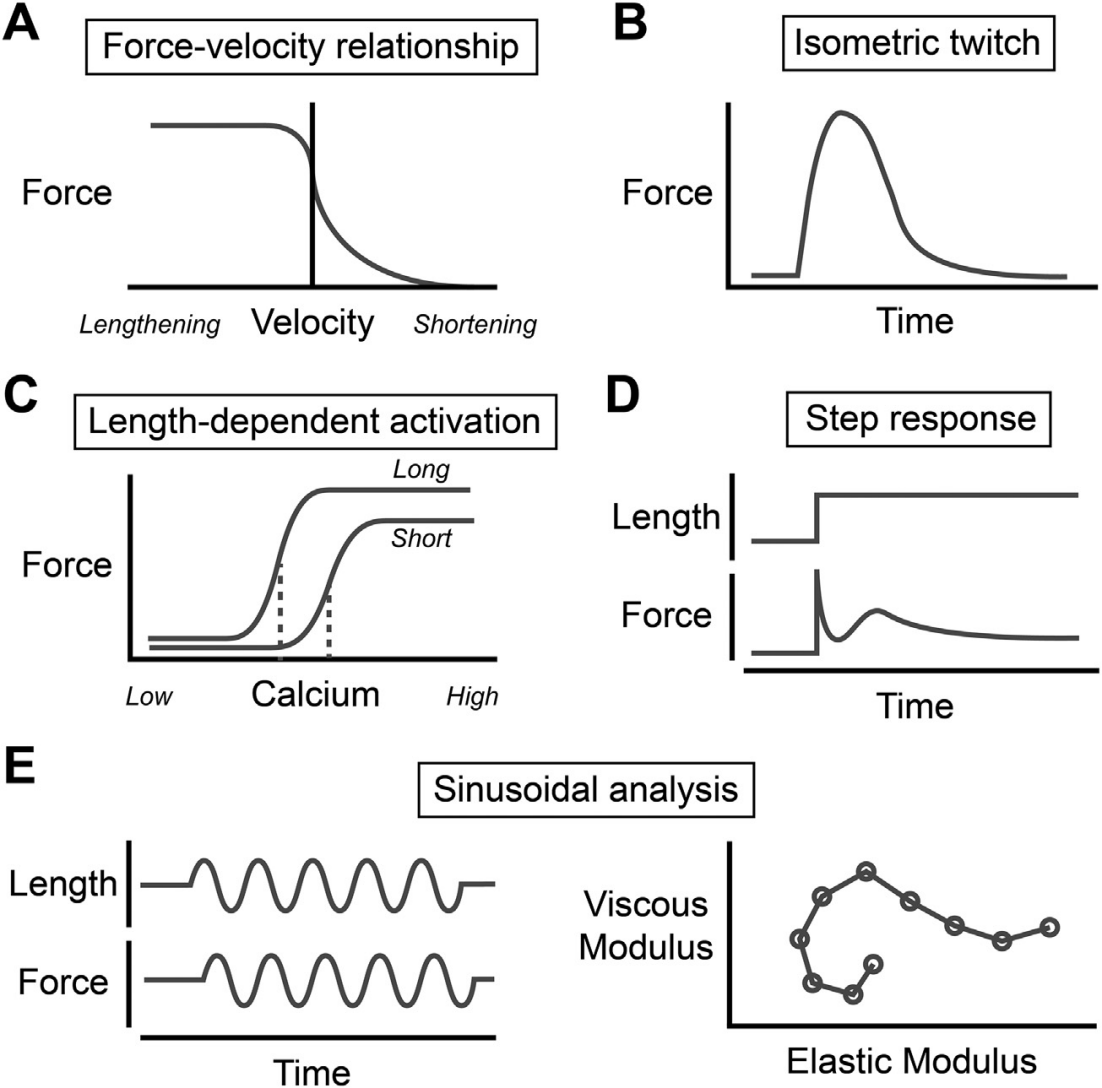
Timelines showing the development of cardiac muscle models in relation to several of the key discoveries in striated and cardiac muscle physiology.

The complex and dynamic material properties of myocardium, in conjunction with the three-dimensional configuration of the myocardial fibres, underpin the complex tissue mechanics of the contracting myocardium [33]. The challenging task of creating models to accurately describe the multivariate properties of myocardial mechanics spans nearly a century (Fig. 2). These efforts have been driven by the opportunity to gain mechanistic insights into cardiac physiology and formulate simulations with sufficient accuracy to find use in clinical applications.

### 1.2 Tissue based models of contraction

Determining the start of any field is contentious. However, the study by A.V. Hill in 1938 [34] described fundamental properties of muscle mechanics that continue to inform muscle physiology studies today. In a series of elegant experiments, Hill related the release of energy by muscle, the mechanical work performed by the muscle, the force of contraction and the velocity of shortening. Hill was able to develop a phenomenological ‘rheological’ model that linked these four variables and derive the characteristic force velocity curve that is now named after him. He also predicted the velocity where peak power will be generated. While the relationship between force and velocity are crucial for muscle physiology, Hill was unable to identify the mechanism that would explain their relationship.

In 1957, Huxley combined recent electron microscopy data with the concept of a crossbridge to develop the first model of tension generation based on protein dynamics [35]. Crossbridges were modelled as bound or unbound with the binding and unbinding rates of crossbridges and actin depending on how far the crossbridge was distorted from its reference state. These spatially varying crossbridge kinetics allowed Huxley's model to recapitulate Hill's force -velocity relationship. The initial model was subsequently extended by Huxley and Simmons [6] to include multiple bound configurations. This allowed the model to reproduce the complex time course of force recovery when a tetanized muscle fiber is subjected to rapid changes in length.

### 1.3 Modelling the biochemistry of contraction

The ability to isolate and measure myosin, actin, ATP and ADP advanced understanding of muscle physiology by allowing the biochemistry of contraction to be studied and subsequently modelled. The initial experiments underpinned enzyme reaction schemes that reflected the biochemical steps of tension generation. Eisenberg and Moos [36] performed a kinetic analysis of actin and heavy meromysoin (the end of the myosin molecule that contains the crossbridge head) and proposed a 4 step cyclical reaction involving actin, myosin and ATP. Lymn and Taylor [37] combined a biochemical description of actin-myosin reactions with a mechanical model of crossbridge force generation to link tension generation with biochemistry. Stein et al., [38] demonstrated that ATP can be metabolised while myosin was still bound to actin. This was inconsistent with the Lymn and Taylor framework. To address this discrepancy Stein et al., proposed a 12 state model of actin – myosin interactions and proposed that crossbridges can bind to actin in multiple states, consistent with the Huxley and Simons model. Stein et al., grouped these states into two sets of crossbridges that were described as strong and weakly bound. Eisenberg et al., [39] expanded the 12 state Stein et al., biochemical actin-myosin model to include velocity dependence and the response to rapid length steps, linking the observations of Hill and Huxley and Simmons to molecular events. A crossbridge kinetic model specific to cardiac myosin isoforms was recently formulated by Mijailovich and coworkers [40].

### 1.4 Modelling the foundations of muscle activation

The insight that free Ca^2+^ activates actin-myosin interaction was first clearly demonstrated by A. Weber in 1959 [9] and subsequently led to new experiments and models. In 1969 Julian proposed a model of transient muscle tension generation [41]. This was an astounding advance given the field's limited knowledge of intracellular Ca^2+^ signalling. Julian's model used a transient activation variable to drive a Huxley model and calculated the distribution of bound crossbridges during a muscle twitch. Greene and Eisenberg [42] observed that crossbridge binding was cooperative and that cooperative activation can occur even in the absence of Ca^2+^. This led them to propose a biochemical crossbridge model that included weakly and strongly bound crossbridges and cooperative binding. Subsequently, Terrell Hill [43] developed a reduced model that represented thin filament Ca^2+^ activation, thin filament cooperativity, crossbridge binding and crossbridge biochemistry and thereby captured many of the processes involved in Ca^2+^-dependent regulation of force.

A crucial addition to understanding and modelling the thin filament was later contributed by McKillop and Geeves, who used biochemical studies of cooperative myosin binding to predict three states of the thin filament regulatory unit (blocked, closed, and open) [44]. A prime example of using model analysis to elucidate molecular mechanisms, their three-state model was subsequently supported by electron microscopy studies that identified three distinct azimuthal positions of tropomyosin corresponding to Ca^2+^-free, Ca^2+^-bound, and myosin-bound conditions [14]. The three-state thin filament regulatory unit model would subsequently be incorporated into numerous models of cardiac muscle contraction.

### 1.5 The early use of models to study cardiac tissue

One of the early applications of mathematical models to study cardiac muscle contraction was performed by Parmley and Sonnenblick [45] in 1967. They used variations of A.V. Hill's rheological model to interpret the passive and active components of cardiac tissue mechanics and deduced properties for these components from measurements of muscle's length-tension relationship. Fung [46] subsequently combined a Hill rheological model, a geometric description of filaments and force-velocity curves to create a model of cardiac muscle tension generation driven by an as yet unknown activation variable.

The development of rapid oscillation techniques in skeletal muscle by Kawai and Brandt [47] allowed the kinetics of muscle biochemistry and muscle mechanics to be studied at the same time. Saeki et al. [48,49] used barium contracture sinusoidal analysis to evaluate two variants of A.V. Hill's rheological model to determine the relationship between passive and active components of cardiac muscle. Sinusoidal analysis was subsequently applied under conditions of differing ATP and inorganic phosphate to link muscle tension dynamics to biochemical reaction rates in a 7 state crossbridge binding model [50]. Finally, Robertson et al., [51] proposed a model of cardiac thin filament activation in the form of Ca^2+^ binding to troponin C. This model was then used extensively for Ca^2+^ troponin C activation in subsequent contraction models.

### 1.6 Modelling thin filament cooperativity

Following observations of thin filament cooperativity [13] and tropomyosin end-to-end polymerization [52], Terrell Hill et al. [53] used the Ising framework to develop a cooperative model of interactions between tropomyosin molecules. This required periodic boundary conditions and a limited number of states. To address these concerns Terrell Hill et al., [54] proposed a more elaborate multi-state cooperative model of actin filament activation and crossbridge biochemistry. Unfortunately, the calculations were too complex to solve with the computing resources available at the time and the work was published as an unsolved theoretical model. Derivatives of these original models would eventually be included in subsequent cardiac mechanics models. Advances in computing power and the use of a cellular automata model allowed Zou and Phillips [55] to simulate tropomyosin neighbour – neighbour interactions and recapitulate the high cooperativity observed in muscle contraction activation. Dobrunz et al., [56] developed a cooperative neighbour-neighbour steady-state model representing explicit regulatory units consisting of 7 actin monomers, a crossbridge, a tropomyosin molecule and the troponin complex. Simulating this system with a simplified network of 9 regulatory units required a system of equations with 262,144 states. A real thin filament, spanning a half sarcomere, has on the order of 26 regulatory units [57] so explicit calculations are likely to overwhelm most computational resources. An alternative was proposed by Smith et al., [58] who represented overlapping tropomyosin molecules as a single continuous chain. The introduction of a bending energy term effectively penalized neighbours with different locations to recapitulate end-to-end cooperativity of tropomyosin. Cooperative models of the thin filament based on tropomyosin overlap were subsequently supported by single-molecule fluorescence studies showing myosin binding in contiguous stretches to regulated actin [59].

### 1.7 Physiological cardiac mechanics sarcomere models

The first model linking Ca^2+^ dynamics to cardiac contraction was published by Panerai in 1980 [60]. They prescribed a Ca^2+^ transient that drove a nonlinear mass action model of actin activation, which was in turn used as an input to a two state crossbridge model. The simulations predicted tension, force-length relationships and oxygen consumption. Guccione and McCulloch [61] developed a cardiac contraction model that represented the known length dependence of tension but moved away from the Huxley crossbridge formulation to provide a model described entirely by ordinary differential equations. This work was paralleled by Landesberg and Sidemen who developed a model of contraction coupled to a model of intracellular Ca^2+^ regulation [62]. In 1998 Hunter et al. [63] published a succinct model of tension generation that combined a biophysical model of thin filament kinetics coupled with a fading memory model of crossbridge kinetics. The Hunter model was one of the earliest designed to drive large deformation mechanics models of tissue and whole heart function. In the following year, Rice et al., [64] published a collection of cardiac contraction models aimed at exploring the different potential roles of thin filament and crossbridge cooperativity in tension generation.

The Rice and Hunter models did not include velocity dependence or represented this dependence with a phenomenological model. Razumova et al., [65] subsequently enhanced a distortion model originally developed by Thorson and White [66] to calculate forces produced by crossbridges in multiple states. The framework could be represented as a system of ordinary differential equations thereby enhancing computational efficiency. Alternative approaches to efficiently represent crossbridge population dynamics have been achieved and used to study the role of tissue strain rate in cardiac twitch relaxation [67].

As models matured there was a growing need to strengthen the links between model parameters, validation, and experimental data. This led Niederer et al., [68] to develop the first contraction model that was based on a set of species and temperature consistent data. These ideas were subsequently extended by Rice et al., [69] who identified the key parameters that could be tuned to create contraction models for different species and distinct experimental conditions. Next, S.G. Campbell built on Eisenberg's research focused on skeletal muscle in the 1980s to create a model of cardiac thin filament cooperativity [70]. Computational cost limited simulations of S.G. Campbell's model to 11 regulatory units but Land and Niederer [71] proposed a variation that included a continuous chain model of tropomyosin and could simulate a full length filament. Recent developments include Land et al.'s data-driven contractile models for human cardiac myocytes, trabeculae and ventricles [72], and Tewari et al.'s simulations that recapitulate the ATP and Pi dependence of dynamic stiffness [73].

Computational cost has also been the main weakness of spatially-explicit models. These simulate contraction by keeping track of the state and location of each molecule in the sarcomeric lattice. To our knowledge, the first spatially-explicit model was developed by Daniel et al. in 1998 [74] but several other groups have now developed similar frameworks [[75], [76], [77]]. One advantage of the approach is that it provides a way of modelling compliant realignment, the vernier-like adjustments in the relative positions of actin and myosin molecules that accompany force development [74]. This is an area of active research and advances in experimental techniques for X-ray diffraction and analysis are providing new insights into the compliance of the thick and thin filaments [78] and potential non-linearities in myosin stiffness [79].

### 1.8 Coupling models of activation to tension generation

An innovative electromechanics model was developed by Kaufmann et al., [80] in 1974 who linked analog circuit models of electrophysiology and tension generation by literally hard-wiring the circuits together. The first coded models either combined simplified Ca^2+^ handling models with complex contraction systems, or complex electrophysiology systems with simplified contraction models [[81], [82], [83]]. Models that integrated detailed electrophysiological and contractile models needed to wait for improvements in computational power. Initial attempts in this area focused on simulating emergent behaviour without strict validation of model predictions [84,85]. Subsequent validated models by S.G. Campbell et al., [86] and Niederer and Smith [87] analysed the effects of Ca^2+^ dynamics and sarcomere heterogeneity, and the slow force response, respectively. Zhang et al., [88] have recently reported a finite element model of the rat left ventricle driven by a detailed sarcomeric model that agreed closely with experimentally measured tissue strains and pressure-volume characteristics. However, such detailed validation is not universal. The number of variables, the disparate data sets and the challenge of model validation has limited the development of this field to date. Many studies now couple previously developed models of electrophysiology and contraction together without model specific validation.

### 1.9 Whole organ modelling

Cardiac mechanics can be approached from the bottom up, scaling from molecules to the heart. An alternative approach is to start at the top by simulating the mechanics of the ventricle. In 1979 Arts et al., [89] proposed an early ventricular model which mimicked transmural variation in fiber orientation using a set of thick walled cylinders. Humphrey and Yin [90] subsequently extended this approach with the first truncated ellipse model of the heart. Guccione et al., [91] returned to modelling the ventricle as a cylinder but introduced the finite element method to solve the large deformation mechanics equations. Anatomical detail was included in a subsequent iteration of this model and Guccione et al., [92] were able to predict strain in a circumferentially symmetric model of the left ventricle and validate their results using experimental data. Nash and Hunter [93], made a significant step forward in whole organ modelling, with the first biventricular model that integrated detailed fiber orientation and biophysical contraction.

Many of these early whole organ models were used to study cardiac physiology. The Arts group developed a truncated ellipse model of left ventricular electromechanics [[94], [95], [96]] that combined a phenomenological contraction model with an Eikonal activation pattern. They used this approach to interpret canine asynchronous activation patterns and to test how modulating the activation would alter whole organ mechanics. More recently, detailed biophysical models of contraction and electrophysiology have been used by Land et al., [[97], [98], [99]] to study the link between sarcomere dynamics and whole organ contraction, and to investigate the impact of eliminating SERCA which plays a crucial role in regulating intracellular Ca^2+^. Finally, Lumens et al., have developed a spectrum of models for simulating cardiac mechanics using a 1-2D representation of the ventricles [100]. Their simplified model captures many of the salient physiological attributes while remaining exceptionally fast to solve. As a result, it is now widely used to help interpret clinical data.

### 1.10 Patient specific models: inferring material properties

One application of cardiac modelling is to deduce material properties from clinical images. Wang et al., [101] demonstrated that this was possible in dogs by calculating passive material properties from MRI data. This approach was subsequently extended to humans by Xi et al., [102,103] using data from two patients with disease and one healthy control. The authors noted that standard constitutive models could not be uniquely constrained by solely comparing predicted and measured displacements. Nasopoulou et al., [104] addressed the problem of identifiability with an energy based cost function and data from seven patients and one control. Gao et al., [105] applied their parameter inference method to three healthy cases. All of these studies used a small number of datasets. Extending these approaches to larger clinical populations remains a significant challenge.

### 1.11 Patient specific models: boundary conditions

As models were improved to reproduce detailed clinical and experimental data there was a need to move from fixed pressure and volume boundary conditions to dynamic models of aortic resistance. Many groups have now adopted the three element Windekessel model that provides a succinct representation of the aortic pressure-flow relationship. Kerkhoffs et al., [106] increased the realism by coupling their bi-ventricle model to a closed loop cardiovascular system model. To capture the interactions between the myocardial wall and blood flow in the cavity, Watanabe et al., [107] developed a complex heart model capturing the electrophysiology, mechanics and chamber fluid dynamics in a single simulation. Nordsletten et al., [108] continued these developments with a fluid-structure-interaction model. Only limited work has been performed to assess the impact of the pericardium [109] and/or the effects of the mitral valve on cardiac mechanics. These are areas for future development.

### 1.12 Modelling specific patients

There is growing use of computer models in clinical applications [110]. 2011 saw the development of electromechanics models of specific patients. Niederer et al., [111] created a model of a patient with left bundle branch block and showed how length dependence of tension generation at the sarcomere scale could play a significant role in the response to cardiac resynchronisation therapy. Aguado-Sierra et al. [112] published a case study demonstrating the creation of a patient specific model, while Sermesant et al. [113] presented a method for developing personalised electro-mechanics models which had been applied to three patients. Crozier et al., [114] created models of three patients and used these to study the relative role of patient physiology and device settings in response to cardiac resynchronisation therapy. Lee et al., [115] then developed the first personalised models that captured patient remodelling in response to therapy to study the effects of pacing location repositioning following CRT device implantation. Okada et al., [116] moved from simulating 1–3 patients to modelling 9 patient hearts, allowing a statistical validation of the virtual cohort. Finally, Kayvanpou et al., [117] created a cohort of 50 patient specific electromechanical models and found a correlation between model parameters and patient response to CRT.

## 2 Discussion

Models of cardiac mechanics have developed from early models of skeletal muscle to cardiac specific models that explicitly represent selected protein-protein interactions. While the development of cardiac models has come a long way there are five gaps identified in this review which require more attention: (a) broadening the sarcomeric proteins included in models, (b) understanding the similarities and differences between modelling frameworks, (c) representing species specificity, and (d) furthering our mechanistic understanding of contraction regulation, and (e) development of detailed organ-scale models.

Cardiac contraction models still contain only a small subset of the proteins present in the sarcomere and omit many of the proteins that are associated with inherited cardiomyopathies. Neither have cardiac contraction models been developed to a point where they can readily simulate pharmacological effects, as has been achieved by the cardiac electrophysiology modelling community [118,119].

In contrast to cardiac electrophysiology modelling [120], there is a lack of consensus on the best modelling framework to simulate the biophysics of the sarcomere. Distinct modelling paradigms have been adopted by different groups so that, for example, a model of tropomyosin kinetics developed in one context has limited equivalence in another modelling framework. Currently three broad modelling approaches are being used: Huxley based partial differential equations, mean field systems of ordinary differential equations, and spatially-explicit models that attempt to represent each protein. The specific contexts where each framework is most relevant and conditions where models are equivalent remains to be determined.

Species specific models are still in early development. The major constituents of cardiac contractile function are consistent across species, however, differences in isoforms and kinetics lead to distinct species specific contraction kinetics (for review see [121]). Two forms of myosin heavy chain are present in mammalian hearts V_1_ and V_3_. V_1_ is a homodimer of two α-MHC molecules and V_3_ is a homodimer of two β-MHC. Mice and rats predominantly express the faster V_1_ or α-MHC, whereas humans and larger animals express predominantly V_3_ or β-MHC [122,123]. Titin content also varies between species with the N2B isoform dominating in rats and mice but the N2BA and N2B titin isoforms being expressed at comparable levels in humans [124]. Differences in the N-terminal of myosin binding protein C are found between species, with smaller animals expressing more proline and alanine residuals [125]. Inclusion of these, and other, species specific isoform differences, along with the distinct differences in calcium handling dynamics between species [8] will be a critical step in the development of models that can be compared directly with experimental data.

Models are inherently approximations that facilitate understanding. Contractile modelling is particularly challenging as the range of measurements needed to constrain the parameters in increasingly complex models are not all performed in the same laboratory, requiring parameters to be fitted to data from multiple groups recorded under different conditions. This means that models are often fitted to distinct permutations of experimental data. In addition, models fitted to different data sets will exhibit distinctly different characteristics, not due to model failure, but due to differences in the underlying data. For example, data fitted to steady state force-calcium relationship from [20] will have fit a single Hill curve with a length dependent cooperativity. In contrast models fit to data from [21] will have a length independent cooperativity but two Hill coefficients corresponding to high and low calcium. Models are also limited by gaps in our understanding of physiological mechanisms. Specifically, models often use phenomenological representations of the length dependence of calcium sensitivity, in part because the molecular mechanisms are incompletely understood [24]. At the same time, as happened with crossbridges, models may generate a testable hypothesis to explain this mechanism.Organ scale models now link down to cellular mechanics and are increasingly tailored to represent the physiology and pathology of specific patients. Early work in organ scale modelling required specific code to solve the equations of large deformation. Now multiple codes are available to solve the standard systems [126]. Cardiac models still face significant computational challenges but key technical questions that also need to be addressed include how to infer model parameters from clinical data, how to incorporate uncertainty and missing clinical data in model creation, and how to represent boundary conditions.

Clinical data are being generated at ever increasing rates. Computer models of cardiac contraction will have an important role to play in integrating and interpreting these data, and to link molecular and cellular observations through to whole organ pump function.
